# Molecular characterization and antimicrobial susceptibility of human *Brucella* in Northeast China

**DOI:** 10.3389/fmicb.2023.1137932

**Published:** 2023-04-14

**Authors:** Han-rui Ma, Hui-jiao Xu, Xin Wang, Zhao-yang Bu, Teng Yao, Zun-rong Zheng, Yang Sun, Xue Ji, Jun Liu

**Affiliations:** ^1^Engineering Research Center of Glycoconjugates, Ministry of Education, School of Life Sciences, Northeast Normal University, Changchun, China; ^2^Changchun Veterinary Research Institute, Chinese Academy of Agricultural Sciences, Changchun, China; ^3^Clinical Laboratory, Beidahuang Group General Hospital, Harbin, China; ^4^Key Laboratory of Jilin Province for Zoonosis Prevention and Control, Changchun, China

**Keywords:** *Brucella melitensis*, whole-genome sequencing, epidemiology, genotyping, antibiotic susceptibility

## Abstract

**Introduction:**

Northeast China has always been an area with severe brucellosis prevalence. This study will identify *Brucella* in Northeast China and test its resistance to antibiotics, in order to clarify its resistance mechanism. *Brucella* is a widespread and highly pathogenic bacteria that poses serious threats to public health and animal husbandry.

**Methods:**

In this study, 61 *Brucella* isolates were identified by abortus-melitensis-ovis-suis polymerase chain reaction (AMOS-PCR) for biotypes and epidemic potential was clarified by multi-locus sequence analysis. Whole-genome sequencing (WGS) was performed and the antibiotic susceptibility of the *Brucella* strains against 13 antibiotics was detected with the use of E-test strips.

**Results:**

The results showed that all of the isolates were *Brucella melitensis* ST8, group CC4 with little genetic variation and obvious geographical characteristics. All 61 *Brucella* isolates were sensitive to doxycycline, tetracycline, minocycline, levofloxacin, ciprofloxacin, gentamicin, and streptomycin, while 24.6%, 86.9%, 65.6%, 27.9%, 3.3%, and 1.6% were resistant to rifampin, azithromycin, cefepime, cefoperazone/sulbactam, cefotaxime, and meperidine/sulfamethoxazole, respectively. This is the first report of cephalosporin-resistant *B. melitensis* in China. The WGS results indicated that about 60% of the antibiotic resistance genes were associated with efflux pumps (mainly the resistance nodulation division family).

**Discussion:**

Brucellosis is usually treated with antibiotics for several months, which can easily lead to the emergence of antibiotic resistance. To ensure the effectiveness and safety of antibiotics for treatment of brucellosis, continuous surveillance of antibiotic susceptibility is especially important.

## 1. Introduction

*Brucella* (phylum *Proteobacteria*, class *Alphaproteobacteria*, order *Rhizobiales*, family *Brucellaceae*) enters the host *via* ingestion, inhalation, or abrasions to the conjunctiva or skin and can survive and multiply in both phagocytic and non-phagocytic cells. Of the 12 known species of *Brucella*, humans are mainly susceptible to only four (*B. melitensis*, *B. suis*, *B. abortus*, and *B. canis*; Franco et al., 2007; Moreno, 2021).

Brucellosis is a zoonotic disease of livestock, leading to abortion or even infertility in females and orchitis in males, which can also pose a threat to human health. The unique pathological features of infected hosts are typically divided into three distinct phases, which include an incubation phase characterized by the lack of clinical symptoms, an acute phase marked by invasion and dissemination of the pathogen in host tissues, and a chronic phase that can eventually result in severe organ damage and death of the host. Nonspecific influenza-like symptoms of brucellosis in humans include pyrexia, diaphoresis, fatigue, anorexia, myalgia, and arthralgia (de Figueiredo et al., 2015). Susceptibility to brucellosis is distinctly linked to occupational exposure, but not sex, age, or season. Brucellosis primarily occurs in countries lacking effective strategies to maintain human and animal health. Endemic brucellosis in provinces and cities in China has been linked to animal husbandry (Lai et al., 2017). According to the Animal Epidemic Prevention Law Press China (2021), brucellosis is classified as a category II disease, with a high incidence throughout the provinces of Inner Mongolia, Heilongjiang, Shanxi, and Jilin (Zhang et al., 2020).

Although several vaccines against brucellosis are available for livestock, there is no effective vaccine for humans, thus human infections are usually treated with combinations of antibiotics. The medication criteria for brucellosis established by the World Health Organization in 2006 recommended doxycycline combined with rifampicin or streptomycin as a first-line therapy and a combination of fluoroquinolone. For children under 8 years of age, pregnant women and patients with co-morbidities, tetracycline, streptomycin, and other drugs with teratogenic potential are prohibited and they are usually treated with rifampicin and third generation cephalosporins. However, the overuse of antibiotics can lead to the emergence of antibiotic resistance. Some strains of *Brucella* are resistant to various antibiotics and the incidence of antibiotic resistance continues to increase annually, especially in the Middle East and North Africa (Trott et al., 2018; Wareth et al., 2022). According to recent data, 100% of *Brucella* isolates in China were resistant to azithromycin, while 62.1%, 58.6%, and 62.1% of isolates in Egypt were resistant to ciprofloxacin, rifampicin, and imipenem, respectively (Liu et al., 2018; Khan et al., 2019; Yuan et al., 2020). However, relatively few studies have investigated the mechanisms underlying the high-rate antibiotic resistance of *Brucella* (Halling and Jensen, 2006; Posadas et al., 2007; Lazaro et al., 2009; Martin et al., 2009). Mechanisms of bacterial resistance mainly include alterations to the targets of antibiotics, inactivation of hydrolase catalysis activity, and the contribution of drug efflux pumps to biofilm formation (Lai et al., 2022). The *Brucella* genome is highly conserved, does not contain plasmids, and there is no evidence of transfer of genetic material, suggesting a relatively small risk of acquired antibiotic resistance (Moreno, 1998; Moreno, 2021).

The incidence of brucellosis has not yet been effectively suppressed and the resistance of *Brucella* to common antibiotics is increasing every year. Molecular epidemiological monitoring of *Brucella* is therefore particularly important. Therefore, the present study aimed to clarify the prevalence of antibiotic resistance in *Brucella* isolates in Northeast China, identify potential genes and mechanisms associated with antibiotic resistance, and provide the experimental data and reference for clinical therapeutics.

## 2. Materials and methods

### 2.1. Bacterial strains

In total, 61 *Brucella* isolates were collected in Northeast China in 2020. *B. melitensis* 16 M, *B. abortus* A19, and *B. suis* S2 were used as positive control strains. *Escherichia coli* ATCC 25922 was used as a quality control strain for testing antimicrobial susceptibility. All experiments were conducted in a biosafety level 3 laboratory.

### 2.2. Identification of *Brucella* strains

Total genomic DNA was extracted using the E.Z.N.A.^®^ Bacterial DNA Kit (Omega Bio-Tek, Inc., Norcross, GA, United States) and abortus-melitensis-ovis-suis (AMOS) polymerase chain reaction (PCR) was performed for species-level identification. Primers designed for amplification of *B. melitensis* 16 M, *B. abortus* A19, and *B. suis* S2 were based on the *Brucella* insert sequence *IS711* (Bricker and Halling, 1994), while general forward and reverse primers for the *Brucella* genus were based on the *Brucella bcsp31* gene sequence (GenBank accession number M20404; Table 1). Every 50 μL PCR assay reaction mixture consisted of 25 μL of 2× PCR premix *Taq* polymerase (CWBio, Beijing, China), 1 μL of the DNA template, 0.5 μL of each primer, and 21 μL of water. PCR amplification consisted of an initial denaturation step at 94°C for 2 min, followed by 30 cycles of denaturation at 94°C for 1 min, annealing at 66°C for 1 min, and extension at 72°C for 1 min. The PCR products were incubated for an additional 5 min at 72°C to facilitate final elongation before storage at 4°C. Finally, the PCR products were separated by electrophoresis using 1% agarose gels (w/v) and sequenced.

**Table 1:** PCR primer sequences.

Primer	Sequence (5′ → 3′)	Amplification product size (bp)
BA	GAC GAA CGG AAT TTT TCC AAT CCC	498
BM	AAA TCG CGT CCT TGC TGG TCT GA	731
BS	GCG CGG TTT TCT GAA GGT TCA GG	285
BG-F	CAA TCT CGG AAC TGG CCA TCT CGA ACG GTA T	208
BG-R	ATG TTA TAG ATG AGG TCG TCC GGC TGC TTG G	
IS	TGC CGA TCA CTT AAG GGC CTT CAT	

BA, *B. abortus* A19; BG-F, *Brucella* genus forward primer; BG-R, *Brucella* genus reverse primer; BM, *B. melitensis* 16 M; BS, *B. suis* S2; IS, *Brucella* insert sequence IS711.

### 2.3. Testing of antimicrobial susceptibility

The antimicrobial susceptibility of the *Brucella* isolates *in vitro* was based on the minimal inhibitory concentration (MIC) of azithromycin, cefoperazone/sulbactam (2/1), cefotaxime, ciprofloxacin, doxycycline, cefepime, gentamicin, levofloxacin, minocycline, rifampicin, streptomycin, trimethoprim/sulfamethoxazole (1/19), and tetracycline, as determined using E-test strips (Liofilchem srl, Roseto degli Abruzzi, Italy) in accordance with the 2020 guidelines of the Clinical and Laboratory Standards Institute (Wayne, 2020). *E. coli* ATCC 25922 was used as a quality control strain. Solutions of the *Brucella* isolates (adjusted turbidity, 0.5) were coated on Mueller–Hinton Agar medium (Beijing Solarbio Science & Technology Co., Ltd., Beijing, China), affixed to an E-test strip, and incubated for 72 h at 37°C. Since the MIC breakpoints of these 13 antibiotics against *Brucella* remain unclear, the MIC breakpoints of *Haemophilus influenzae*, which share similar growth characteristics, were used. After a 72-h incubation period, as determined by the appearance of an even lawn of bacteria, the MIC values were determined as the intersection of the applicable inhibition ellipse displayed by the E-test strips.

### 2.4. Whole-genome sequencing

Whole-genome sequencing (WGS) (Beijing Novogene Bioinformatics Technology Co., Ltd., Beijing, China) consisted of five steps: genome sequencing, genome assembly, genome composition prediction, gene function prediction, and comparative genomics analysis. The harvested DNA was detected by agarose gel electrophoresis and quantified with a Qubit^®^ 2.0 Fluorometer (Thermo Fisher Scientific, Waltham, MA, United States). Sequencing libraries were generated using the NEBNext^®^ Ultra™ DNA Library Prep Kit (New England Biolabs, Ipswich, MA, United States) and index codes were added to attribute each sequence to the appropriate sample. Finally, the PCR products were purified and the size distributions were analyzed to construct libraries with the use of an Agilent 2100 Bioanalyzer (Agilent Technologies, Inc., Santa Clara, CA, United States) and quantified by real-time PCR. The whole genomes of the *Brucella* isolates were sequenced using the NovaSeq 6000 System (Illumina, Inc., San Diego, CA, United States). The original data were filtered for validation. The assembly results obtained from the SOPA denovo genome assembly tool^1^ (Li et al., 2008), the SPAdes genome assembly algorithm^2^ (Bankevich et al., 2012), and ABySS software^3^ (Simpson et al., 2009) were integrated with Contig Integrator for Sequence Assembly of Bacterial Genomes software^4^ (Lin and Liao, 2013). Gaps in the preliminary assembly results were filled using gapclose software (Version: 1.12). Same-lane contamination was removed from the assembly results by filtering reads at a low sequencing depth (average, >0.35). Fragments of >500 bp were removed and potential genes of interest were derived from the final data. Antibiotic resistance genes were identified against the Comprehensive Antibiotic Research Database.^5^

### 2.5. *Brucella* genotyping and grouping

The sequence type of each whole-genome sequence was determined against the Public Databases for Molecular Typing and Microbial Genome Diversity,^6^ as described previously (Li et al., 2008). Nine gene segments with low genetic variation were selected for analysis, which included one intergenic fragment (*int-hyp*), one outer membrane protein gene (*omp25*), and seven housekeeping genes (*aroA*, *cobQ*, *dnaK*, *gap*, *glk*, *gyrB*, and *trpE*). Clonal complexes, groups, and single populations were sorted using the eBURST algorithm.^7^ Two strains with different allele numbers in no more than one of the seven housekeeping genes were considered to belong to the same clonal line.

### 2.6. Phylogenetic analysis

**Figure 1:**
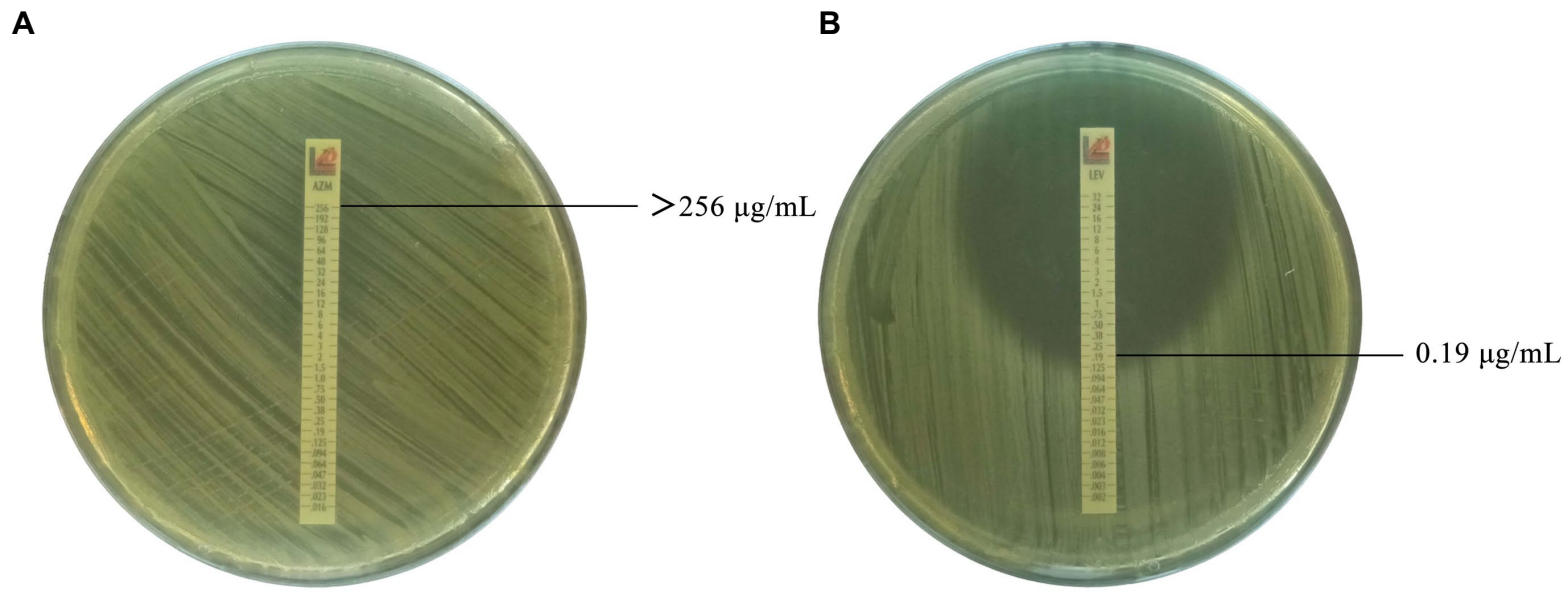
Antimicrobial susceptibility of *Brucella* isolates to cephalosporins (partial). **(A)** Azithromycin against *Brucella* isolate 637 with MIC value of >256 μg/mL. **(B)** Levofloxacin against *Brucella* isolate 637 with MIC value of 0.19 μg/mL.

The evolutionary history was inferred using the neighbor-joining method. An optimal phylogenetic tree with a total branch length of 0.00115159 amino acid substitutions per site is shown in Figure 1. The percentage of replicate trees in which the associated taxa clustered together in the bootstrap test (1,000 replicates) are shown next to the branches. The phylogenetic tree is drawn to scale, with branch lengths in the same units as the evolutionary distances, as calculated by the Poisson correction method. The analysis involved 59 amino acid sequences. All positions containing gaps and missing data were eliminated. The final dataset included 796,249 positions. Evolutionary analyses were conducted using Molecular Evolutionary Genetics Analysis software.^8^

## 3. Results

### 3.1. *Brucella* strains types

**Figure 2:**
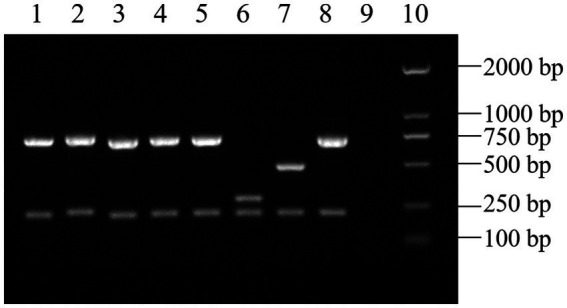
AMOS-PCR results of the *Brucella* isolates (partial). (1–5) *Brucella* isolates. (6) *B. suis* S2. (7) *B. abortus* A19. (8) *B. melitensis* 16 M. (9) Negative control. (10) DL2000 DNA ladder.

As shown in Figure 2, all 61 *Brucella* isolates in this study were identified as *B. melitensis*。 Based on the results of multilocus sequence analysis, all *Brucella* isolates were type ST8, group CC4 and shared nine alleles with no polymorphisms.

### 3.2. Antimicrobial susceptibility testing

All 61 *Brucella* isolates were sensitive to doxycycline, tetracycline, minocycline, levofloxacin, ciprofloxacin, gentamicin, and streptomycin, while 24.6%, 86.9%, 65.6%, 27.9%, 3.3%, and 1.6% were resistant to rifampin, azithromycin, cefepime, cefoperazone/sulbactam, cefotaxime, and meperidine/sulfamethoxazole, respectively. Among the 58 antibiotic-resistant isolates, 31.0% (18/58) were resistant to one class of antibiotics, 67.2% (39/58) were resistant to two classes, and only one was verified as multidrug-resistant (i.e., cephalosporins, macrolides, and antagonists of the folate metabolism pathway; Figure 1; Table 2).

**Table 2:** MIC range and MIC_90_ of 13 antibiotics against 61 *Brucella* isolates.

Antibiotic	MIC (μg/mL)		Classification of isolates (%)			Breakpoints (μg/mL)		
	Range	M_90_	S	I	R	S	I	R
Azithromycin	1–64	64	13.1 (8)	0	86.9 (53)	≤4	–	>4
Cefepime	0.25–2	1	34.4 (21)	0	65.6 (40)	≤2	–	>2
Cefoperazone/sulbactam (2/1)	0.5/0.25–8/4	4/8	72.1 (44)	0	27.9 (17)	≤2	–	>2
Cefotaxime	0.25–2	1	96.7 (59)	0	3.3 (2)	≤2	–	>2
Trimethoprim/sulfamethoxazole (1/19)	1/19–4/76	4/76	98.4 (60)	0	1.6 (1)	≤0.5/9.5	1/19–2/38	>4/76
Rifampin	0.5–2	1	75.4 (46)	24.6 (15)	0	≤1	2	>4
Doxycycline	0.12–0.24	0.12	100 (61)	0		≤1	–	>1
Tetracycline	0.064–1	0.5	100 (61)	0	0	≤2	4	>8
Minocycline	0–0.032	0.032	100 (61)	0	0	≤1	–	>2
Levofloxacin	0.125–1	0.5	100 (61)	0	0	≤2	–	>2
Ciprofloxacin	0.5–1	1	100 (61)	0	0	≤1	–	>1
Gentamicin	0.5–1	1	100 (61)	0	0	≤4	–	>4
Streptomycin	2–4	4	100 (61)	0	0	≤8	–	>8

I, intermediate susceptibility; R, resistant; S, susceptible.

### 3.3. Resistome analysis of the *Brucella* isolates

Analysis of the *Brucella* genome sequences against the Comprehensive Antibiotic Research Database identified 32 antibiotic resistance genes with >40% similarity (Table 3) and 60% associated with three families of efflux pumps [i.e., resistance nodulation division family (RND), major facilitator superfamily (MFS), and adenosine triphosphate-binding cassette superfamily (Kobayashi et al., 2001)].

**Table 3:** Classification of antibiotic resistance genes of *Brucella* isolates (similarity >40%).

Resistance mechanism	Number of genes	Antibiotic-resistant genes
Antibiotic target modifying enzyme	2	*mprF*, 23S rRNA
Antibiotic target replacement protein	2	*dfrE*, *dfrA3*
Antibiotic target protection protein	2	*tetQ*, *mfd*
Antibiotic inactivation enzyme	2	*fosX*, *catB2*
Antibiotic-resistant gene variant or mutant	5	*EF-TU*, *fabI*, *rpoB*, *murA*, *kasA*
Efflux pump complex or subunit conferring antibiotic resistance	19	*mexK*, *qacH*, *golS*, *sav1866*, *macB*, *rosA*, *mdtF*, *rosB*, *msrB*, *mexI*, *triA*, *muxB*, *triB*, *msbA*, *basR*, *bacA*, *vanRI*, *mdtG*, *vanRF*

### 3.4. Phylogenetic tree

**Figure 3:**
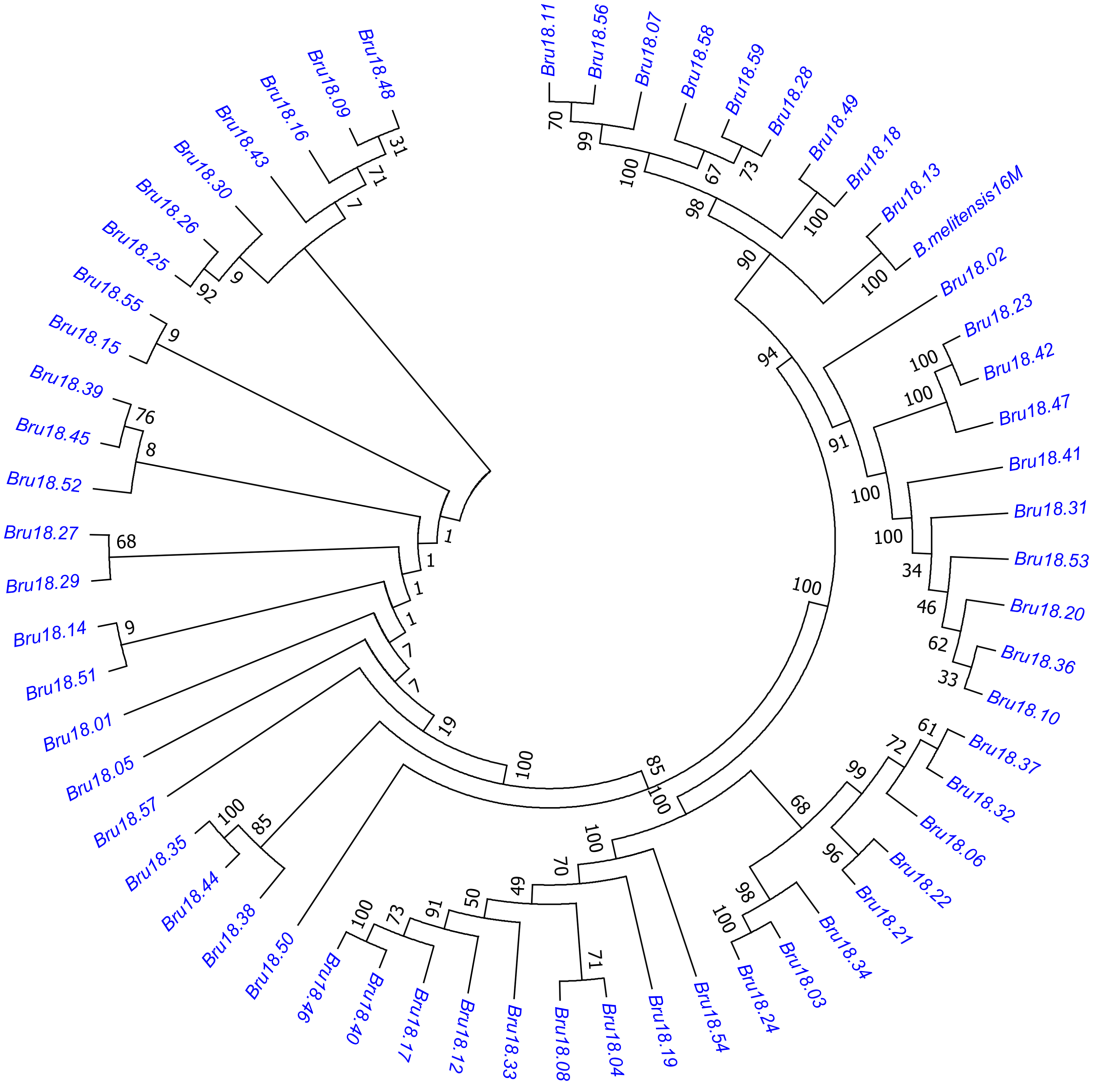
Phylogenetic tree of *Brucella* isolates. The evolutionary history was inferred using the neighbor-joining method and the evolutionary distance was calculated using the Poisson correction method. The evolutionary tree of the relationships among 59 strains of *Brucella* and 16 M can be roughly divided into five small closely related phylogenetic groups with little genetic variation.

A phylogenetic tree was constructed of 59 *Brucella* isolates and *B. melitensis* 16 M. As shown in Figure 3, these isolates can be roughly divided into five small phylogenetic groups with only slight genetic variation of <0.1%.

## 4. Discussion

Brucellosis is endemic throughout Northeast China, which is a major agricultural and pastoral region, thus continuous monitoring is required for prevention and control. In this study, all *Brucella* isolates collected in Northeast China were identified as *B. melitensis* ST8. According to the Public Databases for Molecular Typing and Microbial Genome Diversity website, the most common sequence types of *B. melitensis* are ST8, ST7, and ST11, while ST14, ST17, ST95, and ST96 account for about 7% isolates in Northeast China. *B. melitensis* ST8 has been reported throughout the Eurasian continent, including China, the United Kingdom, Portugal, Norway, Germany, Sudan, Turkey, and India. However, the main epidemic strains and sequence types of *Brucella* vary among different countries, indicating regionality and that transmission might be associated with trade.

The resistance rate of the *Brucella* strains to trimethoprim/sulfamethoxazole determined in this study (1.6%) was notably lower than reported in Ulanqab in south-central Inner Mongolia (7.06%), Xing’anmeng in Inner Mongolia (20%) (Morales-Estrada et al., 2016; Liu et al., 2018; Yuan et al., 2020), while the rate of resistance to azithromycin was similar to that in Ulanqab and Xing’anmeng (100%), suggesting that *Brucella* could gradually become resistant to azithromycin in the near future (Liu et al., 2018; Yuan et al., 2020). In contrast to the results of the present study, isolates collected in Mexico in 2016, Kazakhstan in 2017, and Egypt in 2019 were resistant to ciprofloxacin, streptomycin, rifampicin, and levofloxacin (Morales-Estrada et al., 2016; Shevtsov et al., 2017; Khan et al., 2019). Notably, this study is the first to report cephalosporin-resistant *Brucella* isolates in China and relatively high resistance to cefepime and cefoperazone/sulbactam. As compared to other antibiotics, cephalosporins are more stable and often used administered to children, pregnant women, and patients with complications of brucellosis. However, the emergence of cephalosporins-resistant strains could be challenging for treatment of individuals who are intolerant to first-line antibiotics.

The antimicrobial susceptibility of *B. melitensis* differs among countries, demonstrating regional differences. The similarity of antimicrobial susceptibility between Inner Mongolia and Northeast China indicates that animal trade across regions has led to the spread of *Brucella* and antibiotic resistance. To reduce antibiotic resistance of *Brucella*, it is necessary to provide training and guidance on the use of antibiotics in animal husbandry and conducting regular surveillance.

WGS can help to clarify possible molecular mechanisms underlying antibiotic resistance and identify genes involved in antibiotic transport and inactivation. Previous studies have reported *rpoB* mutations associated with rifampicin resistance of *Brucella* (Marianelli et al., 2004; Valdezate et al., 2010). In the present study, *ropB* mutations accounted for 25% of *Brucella* isolates resistant to rifampicin. In China, the rate of azithromycin resistance of *Brucella* is exceptionally high. Some studies have indicated that the mechanisms underlying resistance of *Brucella* to macrolide antibiotics are related to the RND efflux system, which can be inhibited by the efflux pump inhibitor phenylalanine-arginine β-naphthylamide, and might be responsible for the high rate of azithromycin resistance in this study, similar to the low level of intrinsic antibiotic resistance reported in *Campylobacter* (Corcoran et al., 2006; Halling and Jensen, 2006).

Several efflux pump-related resistance genes were sequenced in this study, which are important mechanisms of intrinsic and partially acquired antibiotic resistance (Li and Nikaido, 2004). Efflux pumps can be divided into five categories: RND, MFS, ABC, small multidrug resistance family, and multi-antimicrobial and toxic compound extrusion (Li and Nikaido, 2009). In the present study, efflux pumps in three families (i.e., RND, ABC, and MFS) were associated with antibiotic resistance. Although relatively few studies have investigated the efflux pumps of *Brucella*, the *bepDE* gene of the RND family in *B. suis* can mediate resistance to tetracycline and fluoroquinolone. In addition, mutations to *bepC* can lead to resistance to fluoroquinolones and aminoglycosides (Posadas et al., 2007; Martin et al., 2009). Therefore, antibiotic resistance of *Brucella* to cephalosporins might be mainly related to efflux pumps, rather than β-lactamase genes. Many studies of mechanisms underlying resistance to cephalosporins have been conducted with *E. coli* and *Salmonella* and found that the antibiotic efflux mechanism was associated with either mutations or deletions of OmpF and OmpC porins, which disrupt the balance between influx and active efflux (Li et al., 2015). The OmpF porin regulates expression of the AcrAB-TolC efflux complex of the RND family (Yoshimura and Nikaido, 1985). Sequencing of genes related to the ABC family of MacAB-TolC efflux pumps found that MacA acts as an adaptor protein that participates in the expression and regulation of the RND (AcrAB-TolC), MFS (EmrAB-TolC), and ABC (MacAB-TolC) efflux pumps (Kobayashi et al., 2001; Beis, 2015). Based on differences in structures and transport mechanisms, specific inhibitors have been developed to target particular efflux pumps. Therefore, inhibitors of efflux pumps and β-lactamase related to cephalosporin resistance can be used to further investigate the mechanisms underlying antibiotic resistance of *Brucella*.

Referring to the WHO guidelines for the treatment of brucellosis and the clinical therapy, the drugs most commonly used are doxycycline, rifampicin, trimethoprim/sulfamethoxazole, and third-generation cephalosporins. Combining 2 or 3 different antibiotics also have a positive effect on success rates in all treatment regimens. According to the results of this study, the clinical treatment was advised to avoid the use of two antibiotics with high resistance rates simultaneously and minimize the use of cefepime and cefoperazone in the selection of cephalosporins. It was also recommended that *Brucella* strains isolated from the patients should be tested for antimicrobial susceptibility before empirical medication to better control the antimicrobial resistance and improve the treatment of infections.

In conclusion, this is the first report of cephalosporin resistance of *Brucella*, indicating that antibiotic resistance has become more prevalent in recent years. Hence, continuous surveillance of antibiotic resistance of *Brucella* should be normalized.

## Data availability statement

The original contributions presented in the study are included in the article/supplementary material, further inquiries can be directed to the corresponding authors.

## Author contributions

XJ and JL conceived and designed the studies. H-jX, Z-rZ, XJ, and JL collected reagents, study materials, and performed the survey. H-rM and XW performed laboratory experiments. Z-yB and TY managed the project. H-rM, XJ, and JL analyzed data and wrote the manuscript. YS, XJ, and JL contributed to the writing and revisions. All authors contributed to the article and approved the submitted version.

## Funding

This work was supported by the Key Science and Technology Projects of the Inner Mongolia Autonomous Region of China (2019ZD006).

## Conflict of interest

The authors declare that the research was conducted in the absence of any commercial or financial relationships that could be construed as a potential conflict of interest.

## Publisher’s note

All claims expressed in this article are solely those of the authors and do not necessarily represent those of their affiliated organizations, or those of the publisher, the editors and the reviewers. Any product that may be evaluated in this article, or claim that may be made by its manufacturer, is not guaranteed or endorsed by the publisher.

## Footnotes

^1^ http://soap.genomics.org.cn/soapdenovo.html

^2^ http://cab.spbu.ru/software/spades/

^3^ http://www.bcgsc.ca/platform/bioinfo/software/abyss

^4^ http://sb.nhri.org.tw/CISA/

^5^ https://card.mcmaster.ca/

^6^ https://pubmlst.org/

^7^ http://eburst.mlst.net/

^8^ https://www.megasoftware.net/
